# Brownian Optogenetic-Noise-Photostimulation on the Brain Amplifies Somatosensory-Evoked Field Potentials

**DOI:** 10.3389/fnins.2017.00464

**Published:** 2017-08-31

**Authors:** Nayeli Huidobro, Abraham Mendez-Fernandez, Ignacio Mendez-Balbuena, Ranier Gutierrez, Rumyana Kristeva, Elias Manjarrez

**Affiliations:** ^1^Integrative Neurophysiology and Neurophysics, Institute of Physiology, Benemérita Universidad Autónoma de Puebla Puebla, Mexico; ^2^Faculty of Psychology, Benemérita Universidad Autónoma de Puebla Puebla, Mexico; ^3^Department of Pharmacology, Centro de Investigación y de Estudios Avanzados, CINVESTAV IPN Mexico City, Mexico; ^4^Department of Neurology, University of Freiburg Freiburg, Germany

**Keywords:** stochastic resonance, optogenetics, neuronal noise, somatosensory cortex, light, photostimulation, neurostimulation, random noise stimulation

## Abstract

Stochastic resonance (SR) is an inherent and counter-intuitive mechanism of signal-to-noise ratio (SNR) facilitation in biological systems associated with the application of an intermediate level of noise. As a first step to investigate in detail this phenomenon in the somatosensory system, here we examined whether the direct application of noisy light on pyramidal neurons from the mouse-barrel cortex expressing a light-gated channel channelrhodopsin-2 (ChR2) can produce facilitation in somatosensory evoked field potentials. Using anesthetized Thy1-ChR2-YFP transgenic mice, and a new neural technology, that we called Brownian optogenetic-noise-photostimulation (BONP), we provide evidence for how BONP directly applied on the barrel cortex modulates the SNR in the amplitude of whisker-evoked field potentials (whisker-EFP). In all transgenic mice, we found that the SNR in the amplitude of whisker-EFP (at 30% of the maximal whisker-EFP) exhibited an inverted U-like shape as a function of the BONP level. As a control, we also applied the same experimental paradigm, but in wild-type mice, as expected, we did not find any facilitation effects. Our results show that the application of an intermediate intensity of BONP on the barrel cortex of ChR2 transgenic mice amplifies the SNR of somatosensory whisker-EFPs. This result may be relevant to explain the improvements found in sensory detection in humans produced by the application of transcranial-random-noise-stimulation (tRNS) on the scalp.

## Introduction

We will start with an explanation of the SR phenomenon because it is a necessary framework to understand our experimental design and results. Briefly, the SR is a phenomenon of nonlinear systems that refers to the increase in the SNR of the output, obtained by an increase in the noise level on the input. Usually, the SNR follows an inverted U-like shape as a function of the input noise level; i.e., there is an intermediate level of input noise for which the SNR exhibits a relative maximum value (Benzi et al., 1981; Douglass et al., 1993; Longtin, 1993; Segundo et al., 1994; Wiesenfeld and Moss, 1995; Collins et al., 1996; Pei et al., 1996; McDonnell and Abbott, 2009). Many psychophysical and electrophysiological experiments in humans and animals support the hypothesis that the brain exhibits this phenomenon (Gluckman et al., 1996, 1998; Simonotto et al., 1997; Winterer et al., 1999; Stacey and Durand, 2000; Zeng et al., 2000; Manjarrez et al., 2002, 2003; Mori and Kai, 2002; Kitajo et al., 2003; Lindner et al., 2004; Long et al., 2004; Moss et al., 2004). Most of the studies about SR in the human brain have employed noisy sensory stimuli to explore their effects on the improvement of sensory perception of the same or other sensory modalities (Manjarrez et al., 2007; Lugo et al., 2008; Mendez-Balbuena et al., 2015; Treviño et al., 2016). In many studies about SR in humans, the brain has been considered as a “black box,” analyzing the external actions of sensory input noise on the behavioral responses. However, recent studies are attempting to open such “black box,” using the application of electrical noise in the brain to enhance tactile sensation in primates (Medina et al., 2012), or tRNS directly on the scalp in humans. The idea behind such pioneering studies is to control the noisy electrical activity of neurons in the cerebral cortex bypassing the noisy sensory stimulation. Such procedure could control the sensory perception and cognition with this type of electrical noise. The tRNS enhances human brain excitability, numerical cognition and facial identity perception (Terney et al., 2008; Cappelletti et al., 2013; Snowball et al., 2013; Romanska et al., 2015). In a more recent study, Van der Groen and Wenderoth (2016) demonstrated that the electrical tRNS of the visual cortex in humans enhance the detection accuracy of visual stimuli. Because such detection accuracy followed an inverted U-like shape, these authors described it as an SR phenomenon. These studies suggested that the electrical tRNS produced the SR via a noisy perturbation of the neuronal membrane potential in the cerebral cortex. It is possible that such type of noise disturbance applied on the scalp in humans may bypass the activation of sensory receptors. Therefore, the next research step, necessary to explore in more detail the impact of noise on the brain over the sensory responses is to employ a new technique to control the neuronal membrane potential in the cerebral cortex. As in the case of the studies by Medina et al. (2012) who employed intracortical stimulation. It is desirable that such new technique could be used to examine the impact of the neuronal noise in the brain on the amplitude of the electrophysiological-evoked sensory responses recorded in the same region of noise stimulation.

The purpose of the present study was to apply for the first time a new technique developed in our laboratory, and which we called “BONP.” In our method, we applied noisy light instead of pulses of light as in the original studies about optogenetics (Boyden et al., 2005). The advantages of this class of stimulation are that it is free of electrical artifacts and it is highly selective. The optogenetic photostimulation activates only the neurons expressing the light-gated opsin channelrhodopsin-2 (ChR2) in cortical layers IV-V in transgenic animals expressing this protein. Such selectivity was demonstrated previously by Wang et al. (2007), in the same type of transgenic mice that we employed in the present study. Furthermore, Arenkiel et al. (2007) found that the Thy1-ChR2-YFP mice, expressed high levels of ChR2-YFP in pyramidal cortical neuros in layer V and also in their apical dendrites projecting into the layer IV. Our experimental design of BONP provides the opportunity to explore the effects of different levels of neuronal noise within the barrel cortex on the SNR of somatosensory evoked field potentials recorded in the same sensory cortex. Our study may contribute to explain the improvements found in sensory detection in primates and humans produced by the application of noisy intracortical microstimulation, or tRNS on the scalp, respectively.

## Methods

We performed experiments in 10 Thy1-ChR2-YFP transgenic mice (line 18) expressing channelrhodopsin-2 under the Thy1 promoter, fused to yellow fluorescent protein (ChR2-YFP) (mean weight 35 ± 3 g). The ChR2 is a cation channel that depolarizes neurons when illuminated with blue light (Wang et al., 2007). Furthermore, we employed other five wild-type littermate mice as a control (weight range 31 ± 3 g). The Thy1-ChR2-YFP transgenic mice (line 18) were obtained from Jackson Labs (JAX USA) and raised in the animal facility of the Centro-de-Investigación-y-de-Estudios-Avanzados CINVESTAV-IPN, México. Polymerase chain reaction (PCR)-based genotyping was done in all the mice. The animals were kept in light and temperature controlled rooms (lights on at 6 a.m. and lights off at 6 p.m.) with free access to food and water. We performed all the experimental procedures following the European Communities Council Directive of 24-November-1986 (86/609/EEC), the guidelines contained in the National Institutes of Health Guide for the Care and Use of Laboratory Animals (85–23, revised in 1985) and the “Norma-Oficial-Mexicana-NOM-062-ZOO-1999.” The protocol was approved by the ethics committee (CICUAL-Proyecto-00489) from the Benemérita Universidad Autónoma de Puebla. The anesthesia consisted of an intraperitoneal injection of a mix of ketamine (90 mg/kg), xylazine (10 mg/kg) and acepromazine (2 mg/kg). To maintain an adequate level of anesthesia, we administered additional doses of this cocktail of ketamine/xylazine/acepromazine every 60–90 min. The mice were completely anesthetized and fixed to a stereotaxic apparatus. The right surface of the skull was exposed and a trephine hole, 7 mm in diameter, was made on the barrel somatosensory cortex, contralateral to the site of whisker stimulation. Then, we carefully removed the dura mater, ~5 mm^2^. A small wall around the aperture in the bone was constructed using dental acrylic, and the space formed was kept filled with mineral oil. Radiant heating and a heating pad were employed to maintain the temperature of the animals at about 37°C.

**Figure 1:**
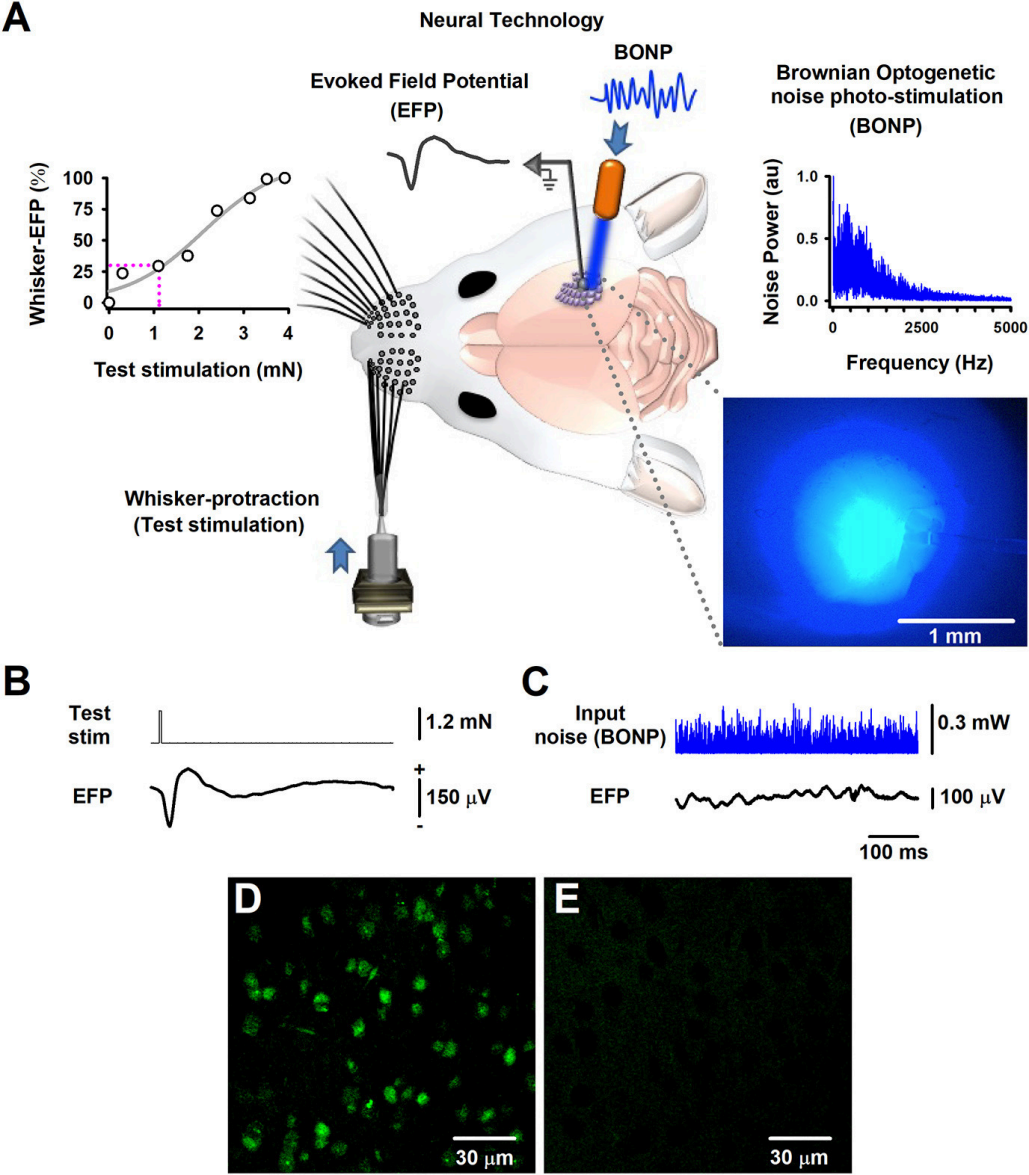
The Brownian-optogenetic-noise-photostimulation (BONP). **(A)** Scheme of the experimental arrangement. Somatosensory neurons from the cerebral cortex were physiologically activated with mechanical protraction of the mouse whiskers (test stimulation pulse) and with BONP (continuous noise). The trace labeled with EFP indicates the Evoked Field potential (EFP) recorded on the surface of the barrel cortex (same scales as in **B**). The upper left panel shows an input-output graph of the intensity of the whisker protraction (test stimulation) vs. the maximal whisker-EFP amplitude. The magenta dashed lines indicate the selected test-stimulus. The upper right panel illustrates the power spectrum of the input noise (Brownian noise) employed in the BONP. The lower right panel shows a picture of the barrel cortex illuminated with the BONP. The light with more intensity covered an area of about 1 mm^2^. **(B)** The whisker-EFP of neurons from the somatosensory cortex responding to whisker protraction (test stim.). **(C)** The same as **(B)** but for the response to continuous BONP (input noise). **(D)** Histological micrograph of neurons expressing ChR2-YFP from the layers IV-V of the somatosensory cortex of a Thy1-ChR2-YFP transgenic mouse. **(E)** The same as **(D)** but for a wild-type mouse. We obtained these micrographs employing the technique of CLARITY and a multiphoton microscope with YFP filter with a 20X water immersion objective.

Mechanical stimulation of the left whiskers allowed the recording of whisker-EFPs on the contralateral barrel cortex. Such stimulation consisted of a whiskers-protraction pulse of 5 ms and 1 Hz (i.e., one brief whiskers-protraction per second), applied using a mechanical stimulator-transducer Chubbuck. The protraction strength was adjusted to produce whisker-EFPs in the right barrel cortex of about 30% of the maximal whisker-EFP (100%). Figure 1A illustrates the experimental arrangement and how the whole bundle of whiskers was stimulated *en masse* to ensure the greatest likelihood of inducing electrical activity (i.e., the whisker-EFPs) in the whole cortical barrels. We recorded EFPs with Ag-AgCl electrodes (200 μm diameter), placed on the surface of the barrel cortex against an indifferent electrode placed on the head muscles.

We recorded the whisker-EFPs in the barrel cortical region in which the averaged EFPs reached their maximal amplitude. These EFPs were amplified with Astromed-Grass amplifiers (filter bandpass, 0.05–30 Hz) and digitized with a Digidata System 1440A (Molecular Devices, Axon Instruments) with a sampling rate of 50 kHz.

The region in which the largest whisker-EFPs occurred, was also continuously illuminated (i.e., stimulated) with the noisy blue light of 470 nm (i.e., the BONP via an optic fiber of 200 μm, with a numerical aperture of 0.39; starter kit from Thorlabs) (Figure 1A). The variation of blue light intensity was Gaussian and controlled via a Wavetake noise generator. We employed an optical power meter PM100D with analog output and sensor type S150C from Thorlabs to characterize the power spectrum of the BONP applied on the brain. The power spectrum of this BONP was similar to the power spectrum of Brownian noise in the range from 0 to 5,000 Hz (upper right graph in Figure 1A). Such noise illumination of blue light over the brain exhibited a flickering appearance with Brownian noise random intensities of light. We employed this type of BONP in the range from 0 to 0.67 mW of optical power. The diameter of the illuminated region of the barrel cortex was about 2 mm when the illumination reached their maximum intensity. Such illuminated area was similar to the whole area of the barrel somatosensory cortex in mice. We employed a Jenoptik camera (model Progres Gryphax Subra) to verify that the light only illuminated the barrel cortex area. The light propagation throughout the brain tissue was attenuated from 1 mm^2^ to up to 2 mm^2^, even when the light was set to their maximal optical power (i.e., 0.67 mW), covering only the barrel cerebral cortex area (blue picture in the right lower panel of Figure 1A). Figure 1A illustrates that the brain area exhibiting the highest intensity of the noisy light was about 1 mm^2^, within the barrel cortex region.

The test stimulation protocol consisted of 32 trials of whiskers mechanical stimulation during zero noise of BONP (control; see the averaged whisker-EFP in Figure 1B) and five different levels of BONP over the barrel cortex (Figure 1C shows an example of a continuous recording of a typical level of input BONP). We continuously applied every level of BONP during the 32 trials of whiskers mechanical stimulation. The application order of the six BONP levels followed a pseudo-randomized fashion. Furthermore, to avoid adaptation, rest intervals of 20 s were included between the noise levels.

Furthermore, we followed the CLARITY protocol (Chung and Deisseroth, 2013) for histology analyses, to obtain high magnification images with a multiphoton laser scanning microscope (Olympus FV1000, Upright BX61WI, YFP filter, 20X water immersion objective) from the “Laboratorio-Nacional-de-Microscopía-Avanzada UNAM, México.” Figure 1D illustrates an image of the somatosensory cortical neurons showing ChR2-YFP expression in the border of layers IV and V of the barrel cortex. The control wild-type mouse did not express ChR2-YFP (Figure 1E).

We recorded whisker-EFPs elicited by different stimulation intensities of the contralateral whiskers as illustrated in Figures 1A,B. The graph in the upper left panel of Figure 1A shows a typical input-output graph for the maximal amplitude of these whisker-EFPs vs. the test stimulus amplitude (strength of whisker protraction in mN). The dashed vertical line in such graph indicates the intensity level employed in the protocols. In 6 transgenic and 5 wild-type mice, we used a test stimulation level which produced EFPs with amplitude of about 30% of the maximal (100%) whisker-EFP amplitude. In other series of 4 experiments (4 other Thy1-ChR2-YFP mice), we employed a test stimulation level which produced EFPs with an amplitude of about 100% of the maximal whisker-EFP amplitude (100%).

We calculated the output SNR as described in **Figure 3** of a previous article from our laboratory (Flores et al., 2016). We computed the output SNR of whiskers-evoked sensory responses in the barrel cortex using the ratio of the rectified whisker-EFP recordings during the application of BONP (S+N) to the rectified EFP recordings during noise conditions alone (N).

We employed the formula:

$$\begin{array}{l} \text{SNR}=|\text{S}+\text{N}|/|\text{N}| \end{array}$$

We calculated this output SNR for every level of BONP applied. Furthermore, we computed the area of the output SNR for all levels of input BONP, including zero noise. Finally, we obtained graphs for the area of the output SNR vs. the input noise (i.e., the BONP).

## Statistical analysis per subject

To test for any statistical difference in the EFP, we considered the 15 normalized areas (NA) of signal-to noise ratio (SNR_NA_) for six levels of BONP. We defined such levels on BONP as ZN: zero noise, N1: first noise level, N2: second noise level, N3: third noise level, N4: fourth noise level and N5: fifth noise level. Because we wanted to compare the conditions ZN vs. N1, ZN vs. N2, ZN vs. N3, ZN vs. N4, ZN vs. N5, and N2 vs. N5 or N3 vs. N5, we performed statistical analysis on these six noise levels. We performed several non-parametric pairwise Signed-Rank Tests to examine the statistical significance of SNR_NA_, between conditions of BONP in all the mice, under the null hypothesis that the differences of the means between conditions ZN vs. N1, ZN vs. N2, ZN vs. 3, ZN vs. N4, ZN vs. N5 and N2 vs. N5 or N3 vs. N5, were zero. Because of multiple comparisons, we used a corrected Bonferroni adjustment. In transgenic mice, all effects are reported as significant if *P* < 0.008. Moreover, in wild-type mice, all results are reported as significant if *P* < 0.01. One-tailed probability was considered for significance.

## Results

**Figure 2:**
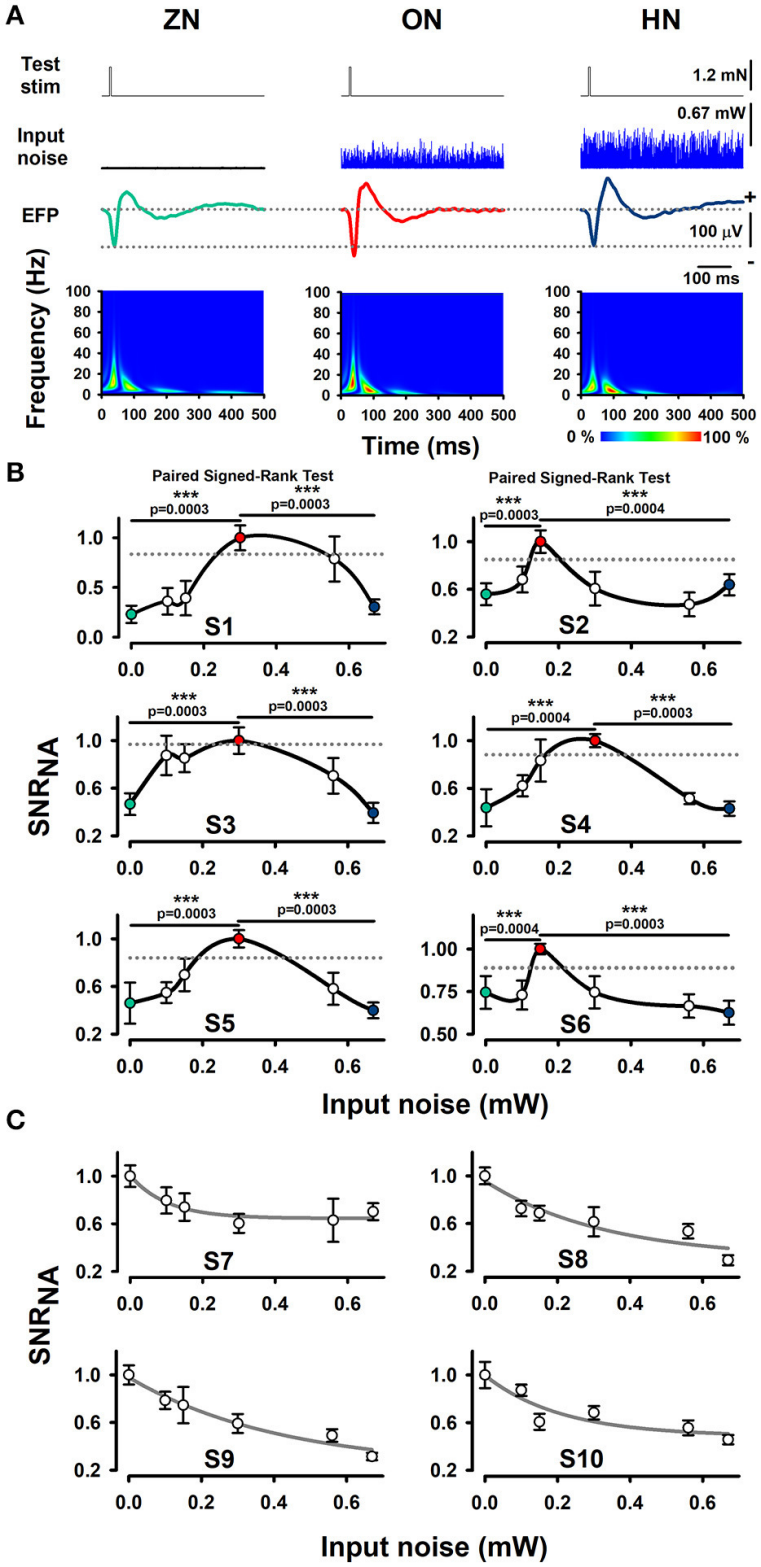
Effect of BONP on the whisker-EFP amplitude and normalized area of SNR of the whisker-EFPs recorded in the somatosensory cortex of Thy1-ChR2-YFP-transgenic mice. **(A)** Grand average of the whisker-EFPs recordings for all the six transgenic mice during three levels of BONP: zero noise (ZN), optimal noise (ON) and high noise (HN). “Test stim” indicates the sub-threshold whiskers-protraction with a constant force of 1.2 mN (see also the dashed lines in the left graph of Figure 1A). Input noise at ZN, ON, and HN is the BONP at three levels of optical power 0.0, 0.3, and 0.67 mW, respectively. The maps in the lower panel illustrate wavelet time-frequency plots for the effect of BONP on the whisker-EFPs illustrated above. **(B)** The normalized area of SNR (SNR_NA_) for the whisker-EFP vs. BONP (input noise in mW) for all the Thy1-ChR2-YFP-transgenic mice (*n* = 6). Note the inverted U-like shape for all these graphs. Here the test-stimulation was applied at 30% of the maximal whisker-EFP amplitude. The dashed horizontal lines represent the magnitude of a 95% confidence interval. **(C)** The same as **(B)** but for test-stimulation at 100 % of the maximal whisker-EFP amplitude in four other Thy1-ChR2-YFP-transgenic mice. ^***^p < 0.001.

Our experimental results show that an intermediate level of optimal BONP increased the mean amplitude of the whisker-EFPs (those elicited with an amplitude of about 30% of the maximal whisker-EFP) for all the six Thy1-ChR2-YFP transgenic mice. Figure 2A shows averaged recordings of the test stimulus (whiskers protraction pulse), BONP (input noise), and the corresponding averaged whisker-EFPs for three conditions: zero BONP (ZN), optimal BONP (ON) and high BONP (HN), obtained from one Thy1-ChR2-YFP transgenic mouse. The graphs in the lower panel of Figure 2A show the corresponding wavelet time-frequency plots for the whisker-EFPs illustrated above.

We computed the SNR area of the whiskers-evoked sensory responses in the barrel cortex using the ratio of the rectified EFP recordings during the application of BONP to the rectified EFP recordings during noise conditions alone (see Methods Section). We calculated the SNR area for every level of noise applied. Figure 2B illustrates the results obtained from six transgenic mice with this method during the different levels of BONP. The abscissa axis of the graphs in Figure 2B indicates the optical-power levels of the BONP in mW units. For clarity, we illustrate with colored circles the ZN (green), ON (red) and the HN (blue). We found that the normalized area of SNR (SNR_NA_) of the whisker-EFPs increased as an inverted U-like shape of the BONP level for all the Thy1-ChR2-YFP transgenic mice. Note that there is an intermediate level of BONP between 0.1 and 0.67 mW (at the tip of the fiber optic) associated with the increase in the SNR_NA_. We examined the statistical significance of our results (Table 1).

**Table 1:** These data show the statistical analysis for the comparison of the effects of BONP in transgenic and wild-type animals.

**Transgenic**	**Signed-rank test**																	
	**ZN–N1**						**ZN–N2**						**ZN–N3**					
	**ZN–Mdn**	**N1–Mdn**	***T***	***z***	***r***	***p***	**ZN–Mdn**	**N2–Mdn**	***T***	***z***	***r***	***p***	**ZN–Mdn**	**N3–Mdn**	***T***	***z***	***r***	***p***
S1	0.22	0.33	24.00	−2.05	−0.53	0.02	0.22	0.35	4.00	−3.18	−0.82	0.0007	0.22	1.00	0.00	−3.41	−0.88	0.0003
S2	0.47	0.61	23.00	−2.10	−0.54	0.018	0.47	1.00	0.00	−3.41	−0.88	0.0003	0.47	0.57	58.00	−0.11	−0.03	0.45
S3	0.44	0.83	0.00	−3.35	−0.86	0.0004	0.44	0.79	0.00	−3.35	−0.87	0.0004	0.44	1.00	0.00	−3.41	−0.88	0.0003
S4	0.37	0.60	21.00	−2.22	−0.57	0.01	0.37	0.95	12.00	−2.73	−0.7	0.003	0.37	1.00	1.00	−3.35	−0.87	0.0004
S5	0.43	0.54	24.00	−2.05	−0.53	0.02	0.43	0.67	13.00	−2.67	−0.69	0.003	0.43	1.00	0.00	−3.41	−0.88	0.0003
S6	0.72	0.76	56.00	−0.23	−0.06	0.41	0.72	1.00	1.00	−3.35	−0.87	0.0004	0.72	0.81	59.00	−0.06	−0.02	0.48
**Transgenic**	**ZN–N4**						**ZN–N5**						**N2–N5, N3–N5**					
	**ZN–Mdn**	**N4–Mdn**	***T***	***z***	***r***	***p***	**ZN–Mdn**	**N5–Mdn**	***T***	***z***	***r***	***p***	**N2, N3–Mdn**	**N5–Mdn**	***T***	***z***	***r***	***p***
S1	0.22	0.78	1.00	−3.35	−0.87	0.0004	0.22	0.28	15.00	−2.56	−0.66	0.005	1.00	0.28	0.00	−3.41	−0.88	0.0003
S2	0.47	0.39	39.00	−1.19	−0.31	0.012	0.47	0.58	19.00	−2.33	−0.60	0.01	1.00	0.58	1.00	−3.35	−0.87	0.0004
S3	0.44	0.65	0.00	−3.35	−0.86	0.0004	0.44	0.37	11.00	−2.78	−0.72	0.005	1.00	0.37	0.00	−3.41	−0.88	0.0003
S4	0.37	0.50	28.00	−1.82	−0.47	0.045	0.37	0.37	47.00	−0.74	−0.14	0.23	1.00	0.37	0.00	−3.41	−0.88	0.0003
S5	0.43	0.55	26.00	−1.93	−0.50	0.027	0.43	0.41	48.00	−0.68	−0.18	0.25	1.00	0.41	0.00	−3.41	−0.88	0.0003
S6	0.72	0.69	29.00	−1.76	0.45	0.039	0.72	0.64	22.00	−2.16	−0.56	0.02	1.00	0.64	0.00	−3.41	−0.88	0.0003
**Wild-type**	**ZN–N1**						**ZN–N2**						**ZN–N3**					
	**ZN–Mdn**	**N1–Mdn**	***T***	***z***	***r***	***p***	**ZN–Mdn**	**N2–Mdn**	***T***	***z***	***r***	***p***	**ZN–Mdn**	**N3–Mdn**	***T***	***z***	***r***	***p***
S1	0.89	0.97	60.00	0.00	0.00	0.5	0.89	0.75	40.00	−1.14	−0.29	0.13	0.89	0.75	43.00	−0.97	−0.25	0.17
S2	0.97	0.91	29.00	−1.48	−0.38	0.07	0.97	0.94	25.00	−0.71	−0.18	0.24	0.97	0.85	15.00	−1.60	−0.41	0.05
S3	0.93	0.87	45.00	−0.85	−0.22	0.19	0.93	0.80	36.00	−1.04	−0.27	0.16	0.93	0.88	44.00	−0.91	−0.23	0.18
S4	0.96	0.98	54.00	−0.34	−0.08	0.37	0.96	0.62	36.00	−1.36	−0.35	0.08	0.96	0.53	36.00	−1.04	−0.27	0.15
S5	0.92	0.80	34.00	−0.80	−0.20	0.21	0.92	0.69	36.00	−1.36	−0.35	0.08	0.92	0.75	28.00	−1.54	−0.39	0.06
**Wild-type**	**ZN–N4**						**ZN–N5**											
	**ZN–Mdn**	**N4–Mdn**	***T***	***z***	***r***	***p***	**ZN–Mdn**	**N5–Mdn**	***T***	***z***	***r***	***p***						
S1	0.89	0.65	45.00	−0.85	−0.22	0.19	0.89	0.65	46.00	−0.80	−0.20	0.21						
S2	0.97	0.84	28.00	−1.22	−0.32	0.11	0.97	0.85	45.00	−0.85	−0.22	0.19						
S3	0.93	0.86	42.50	−1.00	−0.25	0.16	0.93	0.84	45.50	−0.82	−0.21	0.20						
S4	0.96	0.65	36.00	−1.36	−0.35	0.08	0.96	0.34	28.00	−1.54	−0.39	0.06						
S5	0.92	0.95	42.00	−1.02	−0.26	0.15	0.92	0.62	36.00	−1.36	−0.35	0.08						

## Results from the statistical analysis per subject

To examine the statistical significance of SNR_NA_ between six levels of BONP in the whole sample of six transgenic mice, we performed several Signed-Rank tests to compare: ZN vs. N1, ZN vs. N2, ZN vs. 3, ZN vs. N4, ZN vs. N5, and N2 vs. N5 or N3 vs. N5, in each subject. In subjects S2 and S6, the multiple comparisons showed highly significant differences between ZN vs. N2 [S2: ZN (Mdn = 0.47), N2 (Mdn = 1.00), *T* = 0.00, *z* = −3.41, *r* = −0.88, *p* = 0.0003; S6: ZN (Mdn = 0.72), N2 (Mdn = 1.00), *T* = 1.00, *z* = −3.35, *r* = −0.87, *p* = 0.0004], and between N2 vs. N5 [S2: N2 (Mdn = 1.00), N5 (Mdn = 0.58), *T* = 1.00, *z* = −3.35, *r* = −0.87, *p* = 0.0004; S6: N2 (Mdn = 1.00), N5 (Mdn = 0.64), *T* = 0.00, *z* = −3.41, *r* = −0.88, *p* = 0.0003] (see Table 1). Moreover, in subjects S1, S3, S4, and S5, the results uncovered that SNRNA values on N3 were significantly higher than ZN [S1: ZN (Mdn = 0.22), N3 (Mdn = 1.00), *T* = 0.00, *z* = −3.41, *r* = −0.88, *p* = 0.0003; S3: ZN (Mdn = 0.44), N3 (Mdn = 1.00), *T* = 0.00, *z* = −3.41, *r* = −0.88, *p* = 0.0003; S4: ZN (Mdn = 0.37), N3 (Mdn = 1.00), *T* = 1.00, *z* = −3.35, *r* = −0.87, *p* = 0.0004; S5: ZN (Mdn = 0.43), N3 (Mdn = 1.00), *T* = 0.00, *z* = −3.41, *r* = −0.88, *p* = 0.0003]. Similarly, SNR_NA_ values on N3 were significantly higher than N5 [S1: N3 (Mdn = 1.00), N5 (Mdn = 0.28), *T* = 0.00, *z* = −3.41, *r* = −0.88, *p* = 0.0003; S3: N3 (Mdn = 1.00), N5 (Mdn = 0.37), *T* = 0.00, *z* = −3.41, *r* = −0.88, *p* = 0.0003; S4: N3 (Mdn = 1.00), N5 (Mdn = 0.37), *T* = 0.00, *z* = −3.41, *r* = −0.88, *p* = 0.0003; S5: N3 (Mdn = 1.00), N5 (Mdn = 0.41), *T* = 0.00, *z* = −3.41, *r* = −0.88 *p* = 0.0003] (see Table 1). This result demonstrates that the BONP on the barrel cerebral cortex produces an inverted U-like shape in the SNR_NA_ of the whiskers-evoked field potentials of the barrel cortex in Thy1-ChR2-YFP transgenic mice.

**Figure 3:**
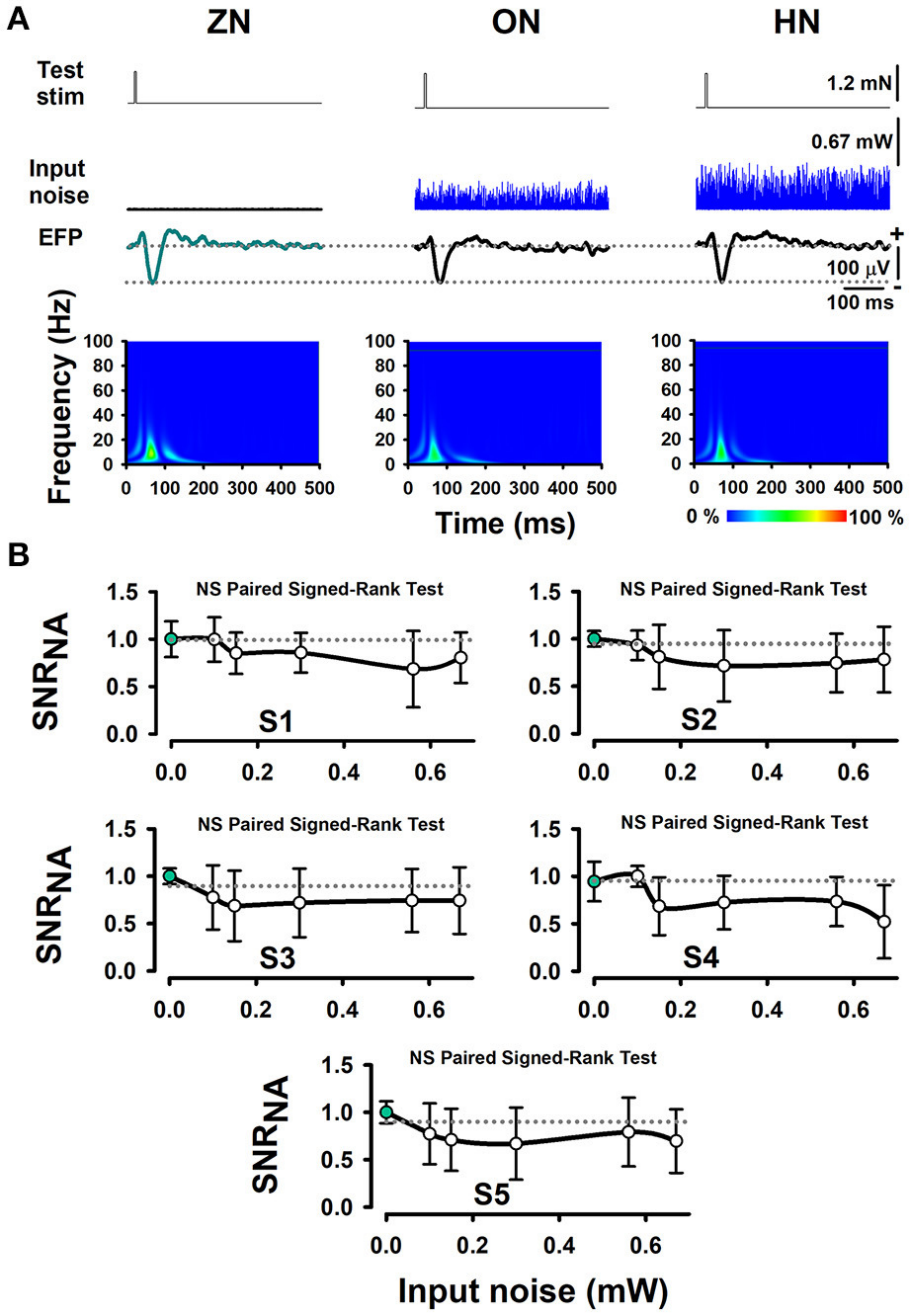
The absence of effects of BONP on the whisker-EFP amplitude and normalized area of SNR_NA_ of the whisker-EFPs recorded in the somatosensory cortex of wild-type mice (the control mice). **(A)** The same format as Figure 2A, but for five wild-type mice. **(B)** The same format as Figure 2B, but for five wild-type mice.

Furthermore, we performed several Signed-Rank tests to examine the statistical significance of the SNR_NA_ in the whole sample of five wild-type mice, between conditions of BONP above mentioned. The results obtained from wild-type mice showed no significant differences (see Table 1 and Figure 3).

In other series of 4 experiments, we analyzed the effects of BONP on the area of SNR_NA_ when the test stimulation level produced EFPs with amplitude of about 100% of the maximal whisker-EFP amplitude. In such case, we observed a decrease in the area of SNR as a function of the input noise intensity. Figure 2C illustrates data from such four mice.

We also performed experiments in Thy1-ChR2-YFP transgenic mice, in which, we applied optogenetic sinusoid photostimulation (at 15 Hz in the same range of intensities as the BONP) instead of BONP. As expected we did not find amplification effects of the whisker-EFPs for such periodic stimulation (data not shown). With this result, we confirm that the SR-like amplification phenomenon of the SNR_NA_ of whisker-EFPs produced by BONP is related to a process of “stochastic facilitation.”

## Discussion

In all Thy1-ChR2-YFP transgenic mice, we observed that an intermediate intensity of BONP of 470 nm applied to the brain increased the SNR of whisker-EFPs (those elicited at 30% of the maximal whisker-EFP amplitude). Conversely, in wild-type mice, we did not observe significant increases in the SNR of the whisker-EFPs when the same levels of BONP were applied.

We employed the BONP protocol to induce noise in neurons. Furthermore, we increased the intensity of the BONP to increase the number of noisy inputs. We found that the graphs of the SNR_NA_ (of whisker-EFPs) vs. the input noise delivered by the BONP followed an inverted U-like shape. A possible interpretation of such results is that the observed amplification in the SNR_NA_ of the whisker-EFP is associated with an SR-like phenomenon. However, further studies are necessary to demonstrate that such inverted U-like shape in the SNR_NA_ is due to stochastic resonance. This study is a first step in the understanding of the impact of BONP in the SNR of sensory evoked field potentials in the brain.

Here we employed the term SR-like (i.e., similar to SR) instead of SR because the definition of SR in populations of neurons in physiological systems is a term of actual debate (McDonnell and Abbott, 2009).

Furthermore, we introduced, for the first time, a method capable of modulating the internal noise in the brain of transgenic animals. These results provide experimental evidence that a controlled modulation of the neuronal noise intensity in the brain produces an SR-like phenomenon in mice expressing the ChR2 opsin sensitive to noisy blue light. Because the activation of ChR2 channels by pulses of light provides controlled changes in the membrane potential of the neurons expressing these channels (Deisseroth, 2015), it is conceivable that also the application of noise light can change the membrane potential of such neurons in a random form. Therefore, we can assume that a possible mechanism for the increase in the SNR of the whisker-EFPs is due to the nonlinear convergent actions of a random membrane potential (i.e., internal noise) and the synaptic inputs from the whisker somatosensory pathway. This idea is consistent with the hypothesis stated by Aihara et al. (2010), which claims that the optimization of the system performance by externally applied noise is related to the interaction between the internal noise of the system and the external noise. The use of intracellular recordings in our experimental paradigm will be necessary to demonstrate this possibility in the ChR2 transgenic mice.

After an inspection of the graphs in Figure 2B, we observed that the intensities of BONP (input noise) necessary to produce an optimal increase of the SNR in the somatosensory EFPs were different in some animals. In some of the animals, the amount of BONP in mW units, necessary to produce an optimal response was below 0.2 mW, but in others above 0.5 mW. We could attribute these differences in the optimal BONP, to individual differences in the excitability state of the barrel cortex neurons during the recordings. Such differences in excitability could be due to the level of anesthesia, the transparency level of the brain to the blue noise light applied, the distribution, and architecture of the dendritic trees of the ChR2 expressing neurons in the barrel cortex, etc. In previous studies in animals (Manjarrez et al., 2002, 2003; Flores et al., 2016) and humans (Manjarrez et al., 2007; Mendez-Balbuena et al., 2015), we also observed this type of differences in the optimal noise level to produce SR among individuals.

Our experimental paradigm of BONP is useful because allow us to modulate the neuronal noise level in the brain directly. Because the BONP uses noise to activate cortical ChR2 neurons, it is tempting to speculate that in the near future it could be employed as the tRNS, to enhance brain excitability, or for the treatment of psychiatric illness. For example, there is compelling evidence that transcranial -electrical or -magnetic stimulation of the orbitofrontal cortex (OFC) in humans produces changes in clinical symptomatology (Fettes et al., 2017). As a first step, it would be possible to design experiments in animal models; for example, to employ the BONP to stimulate the medial OFC, to examine changes in reward learning. This idea is consistent with a recent review by Fettes et al. (2017), who mention that lesions to the medial OFC can impair the animal's ability to associate a previously non-rewarded stimulus with reward. However, we must be cautious when comparing BONP and tRNS. First, because the physiological mechanisms of tRNS in the human brain are still unclear, even that the tRNS is a very powerful method in the clinics and it is getting popular (Antal et al., 2017). Second, because the physiological mechanisms of BONP in the human brain are still completely unknown.

Compared with the noninvasive electrical tRNS employed in humans, our method offers certain advantages. First, the neurons of the ChR2 transgenic animals can selectively be activated by the optical noise stimulation. Conversely, the electrical tRNS stimulates the skin producing the artifactual activation of neurons in the brain innervated by these external inputs from the scalp skin. Second, only the neurons expressing the ChR2 channels sensitive to blue light can be activated by the BONP. In our transgenic mice, these neurons are mostly glutamatergic pyramidal neurons in layers IV and V (Prado et al., 2016). Therefore, we can mainly activate those neurons selectively. Third, with our method of BONP, we can select the diameter of the optic fiber to control the area of stimulation with light in the cerebral cortex. Moreover, we can successfully control the amount of noise delivered to the neural tissue by adjusting the power intensity of the BONP. This simple procedure can allow us to recruit different numbers of neuronal groups that are noisily active. For increased intensities of BONP the number of neurons recruited will increase as well and their random synaptic actions on the neurons producing the sensory EFPs. Fourth, with our method, in future studies, we could express particular noise light-sensitive channels in specific neurons within the brain to explore the effects of internal noise on other sensory responses, both in acute and chronic animals.

However, our experimental paradigm of BONP offers as well the same limitations of the optogenetics technique (Deisseroth, 2015), where the main disadvantage is that for the moment it cannot be applied to the human brain. Another disadvantage of our method of BONP is shared by the electrical tRNS and the optogenetics in chronic animals, as noise power increases, the light or the electrical currents will penetrate deeper into the cortical tissue, and hence the population of cells affected will change. The spatial extent of the stimulated population may also change. Such technical difficulty is a serious problem for all the studies in the field of the optogenetics in chronic animals, in which the population of cells affected by the light can change if different intensities of light are employed. However, this is not a problem for our study given that the barrel cortex exhibits a long-range connectivity along the vertical and horizontal axis (Feldmeyer, 2012). In particular, a long-range connectivity occurs within the cortical layers. In the barrel cortex, there are local connections, intralaminar connections, translaminar connections, and connections between cortical columns (Feldmeyer, 2012). Therefore, the random synaptic actions of the recruited neurons from different layers will produce an unavoidable effect on the neurons producing the sensory EFPs. Based on our results, we could say that it is noteworthy that the SR-like effect in the sensory EFPs is conspicuous, and that it is exhibited independently of the nature of the recruited neurons into the barrel cortex. We could provide a similar explanation for the results of SR obtained from other groups employing electrical tRNS, in which the deep of the spatial extent of the electrically stimulated neuronal populations, may also change (Van der Groen and Wenderoth, 2016). Furthermore, our results are consistent with studies about SR in artificial tactile sensation in primates (Medina et al., 2012), which suggest that the application of noise in the brain could be utilized to enhance prosthetic sensation.

Our stimulation frequencies of higher power are in the range of 500–600 Hz, and they are comparable to those high frequencies employed in electrical tRNS. In this context, our results are also consistent with the observation that transcranial alternating current stimulation (tACS) over the primary somatosensory cortex in different bandwidths allowed that stimulation in Alpha (10–14 Hz) and high Gamma (52–70 Hz) produced a tactile sensation in the contralateral hand (Feurra et al., 2011). We applied Brownian noise of high power in the range from 0 to 1,000 Hz because previous studies (Fertonani et al., 2011) demonstrated that the application of high frequency transcranial random noise stimulation (100–640 Hz) over the visual cortex improved behavioral performance in a visual task. Regarding the type of noise (Brownian) that we applied to the neurons in the barrel cortex, it is possible that other types of noise also could produce similar effects. In fact, it would be interesting to explore the effects of BONP using other colored noises vs. Brownian-noise (Nozaki et al., 1999). Our results suggest that the sensory transmission throughout the barrel cortex was improved because the BONP modulated the endogenous noise in the barrel cortex neurons. In this context, it would be interesting to explore the effects of BONP in transgenic animals, in other superior processing functions that are modulated with tACS, as described in humans (Jausovec et al., 2014). It would also be interesting to explore the effects of bilateral BONP on the cortical inhibition and excitation of the cerebral cortex (Cancelli et al., 2015). Theoretical studies are also consistent with our results, particularly those in which the addition of background noise in populations of modeled neurons (Kawaguchi et al., 2011) or single neurons (Bulsara et al., 1991) can enhance information transmission of subthreshold synaptic inputs.

## Conclusion

The application of an intermediate intensity of BONP in the barrel cortex of ChR2 transgenic mice can significantly amplify the SNR of somatosensory whisker-EFPs.

## Author contributions

EM conceived and designed the experiments and wrote the paper. EM conceived and designed the Brownian optogenetic-noise-photostimulation method. AMF, NH, and EM adapted the noise photostimulation to the *in vivo* setup and performed the experiments. EM, AMF, NH, RK, and IMB performed the analysis of all the experiments. RK and NH performed statistical analysis. RG provided the Thy1-ChR2-YFP and wild-type mice and the starter kit from Thorlabs. All the authors revised and approved the manuscript.

### Conflict of interest statement

The authors declare that the research was conducted in the absence of any commercial or financial relationships that could be construed as a potential conflict of interest.
